# Traumatic brain injury with concomitant injury to the spleen: characteristics and mortality of a high-risk trauma cohort from the TraumaRegister DGU®

**DOI:** 10.1007/s00068-020-01544-5

**Published:** 2020-11-18

**Authors:** Marius Marc-Daniel Mader, Rolf Lefering, Manfred Westphal, Marc Maegele, Patrick Czorlich

**Affiliations:** 1 Department of Neurosurgery, University Medical Center Hamburg-Eppendorf, Martinistraße 52, 20246 Hamburg, Germany; 2 Institute for Stem Cell Biology and Regenerative Medicine, Stanford University School of Medicine, 265 Campus Drive, Stanford, CA 94305 USA; 3 Institute for Research in Operative Medicine (IFOM), University of Witten/Herdecke, Ostmerheimer Strasse 200, 51109 Cologne, Germany; 4 Department of Trauma and Orthopaedic Surgery, University of Witten/Herdecke, Cologne-Merheim Medical Center, Ostmerheimer Strasse 200, 51109 Cologne, Germany

**Keywords:** Traumatic brain injury, Splenectomy, Inflammation, Mortality, Humans

## Abstract

**Purpose:**

Based on the hypothesis that systemic inflammation contributes to secondary injury after initial traumatic brain injury (TBI), this study aims to describe the effect of splenectomy on mortality in trauma patients with TBI and splenic injury.

**Methods:**

A retrospective cohort analysis of patients prospectively registered into the TraumaRegister DGU^®^ (TR-DGU) with TBI (AIS_Head_ ≥ 3) combined with injury to the spleen (AIS_Spleen_ ≥ 1) was conducted. Multivariable logistic regression modeling was performed to adjust for confounding factors and to assess the independent effect of splenectomy on in-hospital mortality.

**Results:**

The cohort consisted of 1114 patients out of which 328 (29.4%) had undergone early splenectomy. Patients with splenectomy demonstrated a higher Injury Severity Score (median: 34 vs. 44, *p* < 0.001) and lower Glasgow Coma Scale (median: 9 vs. 7, *p* = 0.014) upon admission. Splenectomized patients were more frequently hypotensive upon admission (19.8% vs. 38.0%, *p* < 0.001) and in need for blood transfusion (30.3% vs. 61.0%, *p* < 0.001). The mortality was 20.7% in the splenectomy group and 10.3% in the remaining cohort. After adjustment for confounding factors, early splenectomy was not found to exert a significant effect on in-hospital mortality (OR 1.29 (0.67–2.50), *p* = 0.45).

**Conclusion:**

Trauma patients with TBI and spleen injury undergoing splenectomy demonstrate a more severe injury pattern, more compromised hemodynamic status and higher in-hospital mortality than patients without splenectomy. Adjustment for confounding factors reveals that the splenectomy procedure itself is not independently associated with survival.

## Introduction

Traumatic brain injury (TBI) remains a major cause of mortality and disability particularly in young adults [1]. Local and systemic inflammation has been identified as an important contributor to secondary brain injury, which leads to additional downstream damage and represents a potential therapeutic target for neuroprotective approaches [2]. Peripheral immune mechanisms lead to systemic release of proinflammatory mediators and entry of immune cells into the CNS, particularly in the context of blood–brain barrier disruption [3, 4]. Peripheral immune organs like the spleen, which serves as a storage site for immune cells like monocytes [5], may be major contributors to peripheral immune responses. Indeed, a proinflammatory contribution of the spleen has already been demonstrated in the context of cerebral ischemia [6–8]. This important role of the spleen related to systemic immunoresponses has been also extrapolated to experimental TBI [9]. In rodent models of TBI, splenectomy led not only to a suppression of proinflammatory cascades including reduction in inflammatory cytokines like TNF-α and IL-6 but also was associated with better survival along with improved cognitive functions [10, 11]. Of note, experimental data suggest that the adaptive immunity does not seem to considerably contribute to this immune pathomechanism [12]. However, analysis of spleen cells showed an increase in splenic lymphocytes including T cells within 24 h after experimental TBI, which was reversed in the context of CD74 deficiency leading to a smaller lesion size and decreased neurodegeneration [13].

Despite the interesting nature of this preclinical knowledge and the potential therapeutic implications, translational exploration of the effect of splenectomy in patients in a controlled prospective approach appears not feasible. Structured trauma registries may hold the potential to narrow this gap in knowledge by providing relevant data on surgical treatments and outcomes across a range of traumatic impacts. Based on the preclinical data and consequent hypothesis that splenectomy improves survival after TBI, Teixeira and co-workers have queried the National Trauma Data Bank (NTDB) to assess the clinical role of splenectomy in moderate-to-severe TBI patients with splenic injury and exploratory laparotomy [14]. In their study, however, the authors observed an increased mortality with splenectomy, favoring organ preservation over resection in TBI patients with concomitant injury to the spleen. But the validity of this conclusion has been questioned [15]. Potentially confounding factors, such as hemodynamic data, are not routinely captured into the NTDB and could, thus, not be included into the analysis.

This study, therefore, aims to provide a more in-depth description of a trauma cohort suffering both from moderate-to-severe TBI with concomitant spleen injury based on the German TraumaRegister DGU^®^ (TR-DGU). The TR-DGU prospectively collects patient-level data on demographics, injury pattern, comorbidities, pre- and in-hospital management including surgical interventions, e.g. splenectomy, as well as outcomes in four consecutive intervals from the injury until hospital discharge. The assessment of these data, at least retrospectively, allows for systematic analysis of the course of TBI patients with or without splenectomy. Given the previous discrepancy between preclinical and clinical data, the present study is driven by the hypothesis that complete removal of splenic tissue reduces mortality in patients with moderate-to-severe TBI. Accordingly, we provide an observational cohort study with insights on demographics, clinical characteristics including hemodynamic parameters and in-hospital mortality of a selected high-risk cohort of trauma patients with TBI and injury to the spleen. Based on these data, the effect of splenectomy on mortality is tested after adjustment for confounding factors.

## Methods

### TraumaRegister DGU®

The *TraumaRegister DGU*^®^ (TR-DGU) of the *German Trauma Society* (*Deutsche Gesellschaft für Unfallchirurgie*) was founded in 1993. The aim of this multi-center database is a pseudonymized and standardized documentation of severely injured patients. Data are collected prospectively in four consecutive time phases from the site of the injury until discharge from hospital: (A) prehospital phase, (B) emergency room (ER) and initial surgery, (C) intensive care unit (ICU) and (D) discharge. The documentation includes detailed information on demographics, injury pattern, comorbidities, pre- and in-hospital management, ICU course, relevant laboratory findings including data on transfusion and outcome of each individual. The inclusion criterion is admission to hospital via emergency room with vital signs taken, and either subsequent transfer to ICU/intermediate care unit or death before admission to ICU.

The infrastructure for documentation, data management, and data analysis is provided by the *Academy for Trauma Surgery*, a company affiliated to the *German Trauma Society*. The scientific leadership is provided by the *Committee on Emergency Medicine, Intensive Care and Trauma Management* (*Sektion NIS*) of the German Trauma Society. The participating hospitals submit their pseudonymized data into a central database via a web-based application. Scientific data analysis is approved according to a peer review procedure established by *Sektion NIS*. The participating hospitals are primarily located in Germany (90%), but a rising number of hospitals of other countries contribute data as well. Currently, approximately 35,000 cases from almost 700 hospitals are entered into the database per year. Participation in the TR-DGU is voluntary.

There are two datasets that regularly entered into the TR-DGU, a basic dataset and a standard dataset. The basic dataset is mostly provided by smaller hospitals, contains a reduced set of variables and its use is usually restricted to quality assurance. In contrast, the standard and more comprehensive dataset is mostly provided through high-volume and -level trauma centers, contains more in-depth information on management and outcomes and thereby offers unique opportunities for scientific analyses.

The present analysis is in line with the publication guidelines of the TraumaRegister DGU^®^ and registered as TR-DGU project ID 2018-013. Furthermore, the analysis plan was reported to the local ethic committee (WF 059-18).

### Study cohort

**Fig. 1:**
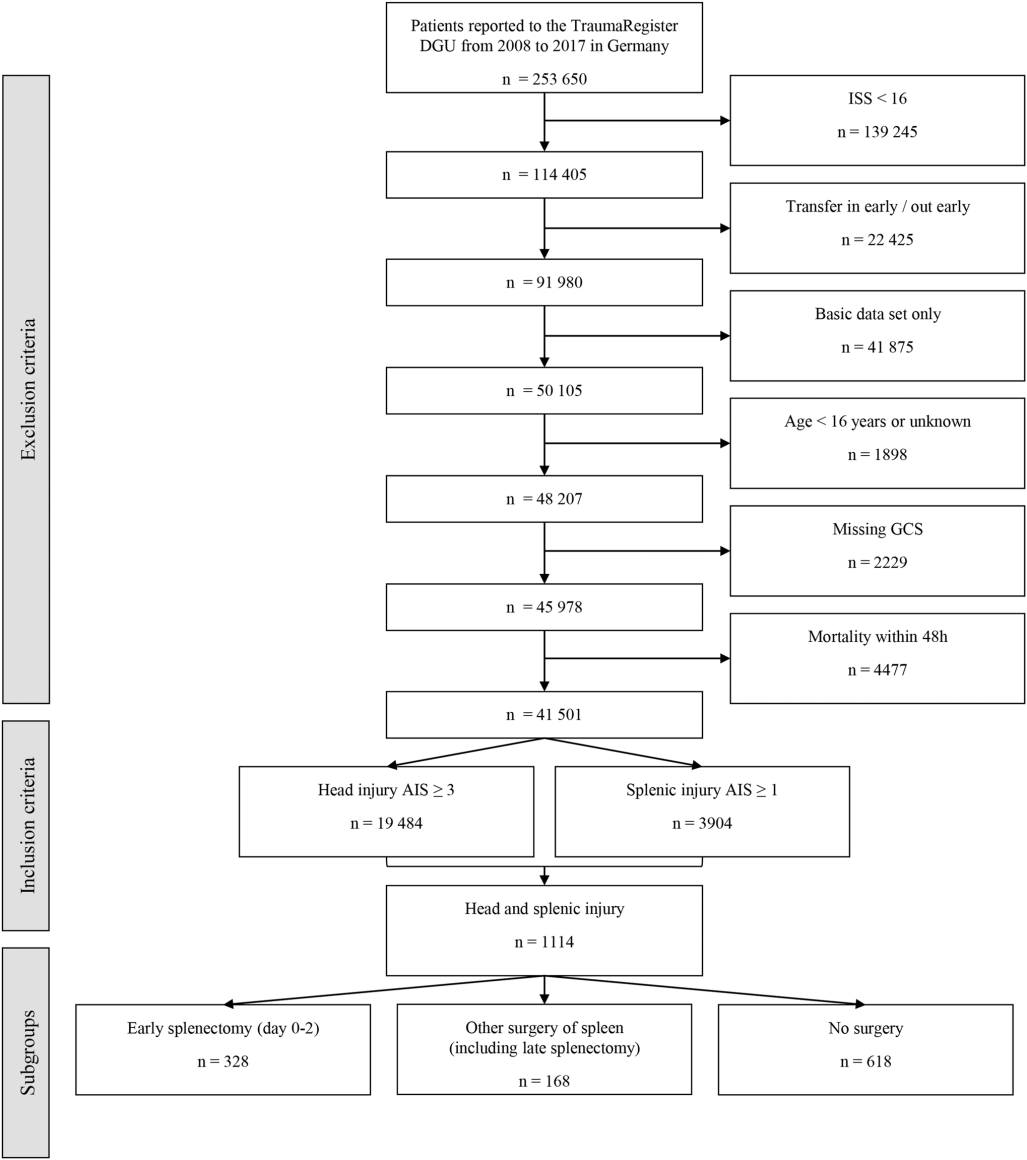
The flowchart describes the exclusion and inclusion criteria as well as relevant subgroups of the study cohort. *AIS* Abbreviated Injury Scale, *DGU* German Trauma Society (Deutsche Gesellschaft für Unfallchirurgie), *GCS* Glasgow Coma Scale, *ISS* Injury Severity Score

Although the TR-DGU database includes a wide range of information for each individual case, only patients ≥ 16 years of age with an ISS ≥ 16, moderate-to-severe TBI (defined as an Abbreviated Injury Scale (AIS) of the head score of ≥ 3) and any abdominal injury (AIS_Abdomen_ ≥ 1) treated between 2008 and 2017 in Germany with complete status documentation of Glasgow Coma Scale (GCS) and surgical procedures were included in this study. Data analysis is based on the yearly registry download (performed on May 31, 2018) for all completely documented patients admitted until December 31, 2017. The total number of patients documented in TR-DGU in the specified period (2008–2017) was 289,698. Patients with basic data set documentation were excluded a priori. Likewise, early secondary transfers (< 48 h) to a different hospital were also not considered as no outcome information for these patients is available from the TR-DGU database. Similar to Teixeira et al. [14] patients who died within 48 h after admission were excluded for two main reasons. First, in order to control for the self-fulfilling prophecy effect of initial withdrawal of care. Second, since the hypothesis of this study is built on the assumption of a proinflammatory effect of splenic tissue, TBI injury pattern leading to death within the first two days was not considered as an adequate basis to elucidate neuroinflammatory mechanisms of secondary brain injury. These cases demonstrate already a maximal primary injury severity as well as only limited time for inflammatory mechanisms to develop. Patients with splenic injury were grouped whether an early splenectomy (day 0–2) was performed or not. Figure 1 provides an overview about the selection process.

### Variables

The primary outcome parameter was in-hospital mortality. Secondary outcome parameters were early neurologic outcome at discharge, ICU length of stay and hospital length of stay. Early neurologic outcome at discharge as assessed by the Outcome Scale (OS) of the TR-DGU which is derived from the Glasgow Outcome Scale. The OS consists of five levels: death (1), persistent vegetative state (2), severe disability (3), moderate disability (4), and good recovery (5) [16]. An OS of 4 and 5 was defined as favorable early neurologic outcome.

Variables extracted from the TR-DGU included basic demographic data, trauma mechanism and American Society of Anesthesiologists (ASA) Physical Status Classification. Parameters of trauma severity were Injury Severity Score (ISS), AIS of different body regions and the Revised Injury Severity Classification, version II (RISC-II) predicting the risk of death [17, 18]. The RISC-II score has been validated for mortality prediction depending on the clinical status in the emergency room of a large number of patients included into the TraumaRegister DGU^®^ data set [18]. It considers the AIS severity level of worst, second-worst injury and head injury as well as the variables age, sex, pupil reactivity and size, pre-injury health status, blood pressure, acidosis, coagulation, hemoglobin and cardiopulmonary resuscitation (CPR). The RISC-II score (higher value means better survival) is transformed into a risk of death estimator using the logistic function. Additional variables extracted included GCS, intubation, transfusion of packed red blood cells (RBCs) or volume in the preclinical or ER setting. Further parameters of hemodynamic status were systolic blood pressure on scene and at admission also dichotomized using 90 mmHg as a threshold of hypotension, initial base deficit and initial hemoglobin concentration (Hb). Moreover, time of splenectomy was included.

### Statistical methods

Statistical analyses were performed using SPSS statistical software (SPSS Version 24.0, IBM Inc., Armonk, New York, USA). Data are presented as mean ± standard deviation (SD) for continuous variables, as median and interquartile range (IQR) for ISS and GCS, and as numbers and/or percentages for categorical variables. Univariate analysis was performed with chi-square test for counts and Mann–Whitney *U* Test for measurements. To assess the independent impact of splenectomy on in-hospital mortality, a multivariable logistic regression analysis was performed. The model was adjusted for the severity of splenic injury (AIS_spleen_), other surgical interventions of the spleen, and the RISC-II score. Regression coefficients are presented together with the respective odds ratio (OR) and corresponding 95% confidence interval (CI).

In patients with surgical therapy, we additionally performed a propensity score analysis with splenectomy as dependent variable. Independent predictors were: ISS, severity of head injury, severity of splenic injury, age, sex, blood transfusion, mass transfusion, penetrating injury, unconsciousness, and hospital level of care. Patients with and without splenectomy were then matched according to the propensity score (± 1%).

## Results

The study cohort consisted of 1114 patients who met the set inclusion criteria (Fig. 1). Out of these, 328 (29.4%) had undergone early splenectomy. Among the remaining 786 patients were 168 patients (15.1%) who received either a spleen preserving intervention, or a late splenectomy (*n* = 13). Table 1 displays the rate of splenectomy against the magnitude of injury to the spleen reflected by the AIS_spleen_. The majority of splenectomies (95.9%) were performed within two days after initial trauma (Table 2).

**Table 1:** Rate of splenectomy in relation to the severity of splenic injury, graded according to AIS

Spleen AIS	No/late splenectomy	Splenectomy	Total
2	529 (98.3%)	9 (1.7%)	538
3	209 (71.6%)	83 (28.4%)	292
4	36 (18.3%)	161 (81.7%)	197
5	12 (13.8%)	75 (86.2%)	87
Total	786 (70.6%)	328 (29.4%)	1114

*AIS* Abbreviated Injury Scale

**Table 2:** Timing of splenectomy

Day	*n*	%	
0	300	87.7	Early splenectomy 95.9%
1	25	7.3	
2	3	0.9	
3–4	8	2.3	Late splenectomy 4.1%
> 4	6	1.8	

The groups shared a male-dominant sex distribution (about 70%) and a similar mean age (about 40 years). The most common trauma mechanism was traffic related (75.2%) and the most common concomitant injury was thoracic trauma. The percentage of patients with an initial GCS ≤ 8 was higher in patients who were splenectomized (49.4% vs. 57.3%, *p* = 0.017). Patients with early splenectomy had a much higher Injury Severity Score (median: 34 vs. 44, *p* < 0.001) and a worse RISC-II prognosis (32.7% vs. 20.8%, *p* < 0.001). These patients were also more frequently hypotensive upon admission (19.8% vs. 38.0%, p < 0.001) and in need for blood transfusion (30.3% vs. 61.0%, *p* < 0.001).

The mortality was 20.7% in the splenectomy group and 10.3% in the control group without early splenectomy. Crude neurological outcome at discharge was documented as favorable in 65.1% of splenectomized survivors and in 71.1% of control cases (*p* = 0.083). The median length of stay (LOS) in hospital was similar in both groups (23 vs. 25 days, *p* = 0.25) but median ICU stay (14 vs. 16 days, *p* = 0.014) and duration of mechanical ventilation (7 vs. 14 days, *p* < 0.001) were longer in the splenectomized group. Table 3 provides an overview about the demographic, clinical and outcome data of the two subgroups.

**Table 3:** Demographic, clinical and outcome data of patients with and without splenic injury and splenectomy

Variable	Category	No or late splenectomy *n* = 786	Early splenectomy *n* = 328	*p* value
Sex	Male	559 (71.5%)	221 (67.6%)	0.20
Age [years]	Mean ± SD	40.1 ± 20.1	39.6 ± 19.3	0.82
Trauma mechanism	Traffic accident	587 (75.2%)	242 (75.2%)	0.39
	High fall	134 (17.2%)	65 (20.2%)	
	Low fall	40 (5.1%)	8 (2.5%)	
Mechanism	Blunt	748 (98.7%)	309 (97.8%)	0.28
Physical status	ASA 3&4	64 (9.9%)	22 (9.2%)	0.73
ISS	Median (IQR)	34 (27–43)	44 (41–57)	< 0.001
Expected mortality (RISC-II)	Mean [%]	20.8	32.7	< 0.001
AIS Head	3	351 (44.7%)	136 (41.5%)	0.70
	4	263 (33.5%)	113 (34.5%)	
	5/6	172 (21.9%)	79 (24.1%)	
Other relevant abdominal injury	AIS higher than spleen	78 (9.9%)	15 (4.6%)	0.003
Thorax	AIS ≥ 2	677 (86.1%)	295 (89.9%)	0.082
Spine	AIS ≥ 2	330 (42.0%)	146 (44.5%)	0.44
Upper extremity	AIS ≥ 2	395 (50.3%)	160 (48.8%)	0.65
Lower extremity	AIS ≥ 2	270 (34.4%)	124 (37.8%)	0.27
Pelvic fracture	AIS ≥ 2	259 (33.0%)	136 (41.5%)	0.007
GCS	Median (IQR)	9 (3–14)	7 (3–13)	0.014
Unconscious	GCS ≤ 8	384 (49.4%)	185 (57.3%)	0.017
Intubated on scene	Done	536 (67.8%)	241 (76.3%)	0.005
CPR	Done	24 (3.0%)	15 (4.7%)	0.16
Hypotension on scene	sysBP ≤ 90 mmHg	152 (21.1%)	104 (36.0%)	< 0.001
sysBP on scene	mean ± SD [mmHg]	118 ± 31	107 ± 34	< 0.001
Hypotension at admission	sysBP ≤ 90 mmHg	146 (19.8%)	117 (38.0%)	< 0.001
Blood transfusion	Done	238 (30.3%)	200 (61.0%)	< 0.001
Number of pRBC (if transfused)	Median (IQR)	4 (2–8)	7.5 (4–14)	< 0.001
Volume (prehospital)	Median (IQR) [ml]	1000 (500–1500)	1275 (1000–2000)	< 0.001
Volume (ER)	Median (IQR)	1000 500–2500	2000 (1000–3625)	< 0.001
Base excess	Mean ± SD [mmol/l]	− 3.6 ± 4.8	− 5.2 ± 5.5	< 0.001
Hemoglobin	Mean ± SD [g/dl]	11.8 ± 2.6	10.4 ± 2.9	< 0.001
Mortality	In hospital	81 (10.3%)	68 (20.7%)	< 0.001
Outcome scale (survivor only)	2—PVS	50 (7.2%)	24 (9.8%)	0.22
	3—severe	152 (21.7%)	62 (25.2%)	
	4—moderate	254 (36.3%)	89 (36.2%)	
	5—good	243 (34.8%)	71 (28.9%)	
Days on ICU	Median (IQR)	14 (6–25)	16 (8–28)	0.014
Days in hospital	Median (IQR)	23 (14–34)	25 (15–39)	0.25
Duration of mechanical ventilation (days)	Median (IQR)	7 (2–17)	10 (4–20)	< 0.001

*AIS* Abbreviated Injury Scale, *ASA* American Society of Anesthesiologists Physical Status Classification, *CPR* cardiopulmonary resuscitation, *ER* emergency room, *GCS* Glasgow Coma Scale, *ICU* intensive care unit, *IQR* interquartile range, *pRBC* packed red blood cells, *RISC-II* Revised Injury Severity Classification, version II, *sysBP* systolic blood pressure, *ISS* Injury Severity Score, *SD* standard deviation, *PVS* persistent vegetative state

After adjustment for the RISC-II score, injury severity of the spleen, and other surgical interventions in a logistic regression model (Table 4), splenectomy was not found to exert an effect on in-hospital mortality (OR 1.29 (0.67–2.50), *p* = 0.45). RISC-II score was the only covariable demonstrating a significant effect in this model.

**Table 4:** Multivariable regression analysis for dependent variable in-hospital mortality (1114 patients)

	Coefficient	*p* value	OR	95% CI of OR
RISC-II score	− 0.70	< 0.001	0.50	0.44–0.55
AIS_spleen_ (3)*	0.10	0.74	1.10	0.62–1.96
AIS_spleen_ (4/5*)	0.13	0.71	1.14	0.56–2.32
Splenectomy*	0.25	0.45	1.29	0.67–2.50
Other surgery*	− 0.17	0.61	0.84	0.44–1.62
Constant	− 1.39	< 0.001		

*AIS* Abbreviated Injury Scale, *CI* 95% confidence interval, *OR* odds ratio, *RISC-II* Revised Injury Severity Classification, version II

*Reference group for AIS spleen: AIS 2; reference group for surgery: conservative treatment

The sub-cohort of patients with surgical therapy related to the spleen was further described with a propensity score analysis. Within this sub-cohort, 107 patients with and without splenectomy, respectively, could be matched according to the propensity score. No statistical differences in mortality or secondary outcome parameters between the two groups were observed (Table 5).

**Table 5:** Propensity score analysis for patients with surgical therapy

	Spleen-preserving surgery	Splenectomy	*p* value
No. of patients	107	107	
Probability for splenectomy (propensity score)	59.3%	59.3%	1.00
Age (years)	37 (19)	39 (19)	0.39
Injury Severity Score	42 (11)	42 (12)	0.73
Mortality	12.1%	16.8%	0.44
Multiple organ failure	61.0%	60.2%	1.00
Sepsis	16.2%	18.6%	0.71
Good outcome (OS 4–5)	67.3%	58.1%	0.20
Days on intensive care unit	15 (7–24)	17 (9–29)	0.35
Days in hospital	24 (16–41)	27 (17–40)	0.39

A total of 214 patients with and without splenectomy were matched according to the propensity score. Statistical tests: Fisher’s exact test for counts; Mann–Whitney *U* Test for measures

## Discussion

The spleen is a major lymphatic organ serving as a storage site for lymphocytes as well as monocytes, which can rapidly be deployed to tissue injuries [5, 13]. A protective effect of splenectomy against ischemic damage and associated proinflammatory reactions in different organs has already been described [19, 20]. With regards to cerebral injuries, an inflammatory involvement of the spleen in the pathophysiology of ischemic brain lesions has been demonstrated [8, 21]. Activated spleen cells have been shown to secrete enhanced levels of inflammatory mediators within the first 22 h after experimental stroke [22]. However, it has also been demonstrated that this is followed by splenic atrophy with a concomitant state of immunosuppression four days after stroke induction [23]. Splenectomy prior or splenic irradiation after experimental stroke induction reduced infarction volumes and cerebral immune cell counts in rats [6, 7]. Investigation of the role of splenectomy in experimental TBI showed similar findings of an ameliorated peripheral inflammatory reaction [9–11]. Immediate posttraumatic splenectomy led to an improved survival and cognitive outcome in rats [10]. The neuroprotective effect of splenectomy has been associated with a suppression of the proinflammatory mitogen-activated protein kinase signaling pathway in experimental TBI [11]. In summary, these preclinical findings showed promising potential of splenectomy to improve secondary brain injury by suppression of inflammation after acute cerebral injury. Moreover, splenectomy may also imply an earlier and more definitive bleeding control preventing a scenario of hemorrhagic shock due to occult hemorrhage, which is detrimental in the setting of polytrauma [24].

Besides the proinflammatory role of the spleen after TBI, a cholinergic anti-inflammatory pathway leading to spleen-dependent immunosuppression and nosocomial infections, particularly pneumonia, after TBI has also been described [25]. However, a recent clinical study contradicted this mechanism in humans by showing that splenectomy did not mitigate the risk of pneumonia in TBI patients [26]. This also underlines the importance of translationally confirming preclinical with clinical findings to further elucidate the actual human pathophysiology.

In the present study, we describe a trauma cohort with moderate-to-severe TBI and concomitant splenic injury. The total rate of splenectomy was 29.4% and markedly increased with higher magnitude of injury as reflected by the AIS_spleen_. Congruent with this high rate of splenectomy in AIS_spleen_ 4&5 patients, TBI has previously been identified as a predictor for failure of nonoperative management in high grade splenic injury [27]. Splenectomized patients were about twice as likely to receive RBC transfusions as compared to the overall study cohort. Besides indicating a hemodynamically more unstable group, the role of RBC transfusion in the setting of TBI itself remains ambiguous with particularly liberal transfusion management being associated with worse outcomes [28, 29]. In general, patients who had received an early splenectomy were more severely injured and showed worse crude outcomes. This observation corresponds to the work by Teixeira et al. [14]. In their work based upon NTDB data, splenectomy remained associated with higher mortality also after adjustment for variables including age, hypotension at admission, GCS, AIS_abdomen_, ISS, solid-organ or hollow-viscus injury and grade of splenic injury. Based on our logistic regression model and study cohort, we were not able to show a beneficial effect of splenectomy on outcome either. But in contrast to the NTDB analysis, we did not observe a negative effect on in-hospital mortality associated with splenectomy either. A possible reason might be different inclusion criteria given that only patients who had received an exploratory laparotomy were included in the study by Teixeira et al. [14]. However, we did not detect a significant difference in outcome in a propensity score subgroup analysis between patients with splenectomy or other splenic surgery either. Moreover, the difference in the effect of splenectomy might also be attributed to usage of the RISC-II score for adjustment. The RISC-II score allows a comprehensive characterization of the individual patient at the time of admission [18]. Similar to the regression model of Teixeira et al*.*, it also considers the variables: age, blood pressure and overall trauma severity scales (AIS severity level of worst, second-worst and head injury). In addition to the motor function of GCS, pupil status is included as a further TBI-specific clinical sign. Additionally, besides pre-injury health status, important circulation specific parameters such as acidosis, coagulation, hemoglobin and CPR are included as well. A later deterioration, however, is not covered by the RISC-II and thus, splenectomy might be an indicator for such a dynamic. Thus, the conflicting findings from two studies can be attributed to insufficient patient adjustment by variables not considered or available in the NTDB dataset particularly displaying hemodynamic instability, which represents a major aspect in patients with severe splenic injury [15]. In this context, hypotension has been demonstrated to be predictive of mortality and functional outcome in TBI, and adherence of relatively high thresholds was associated with favorable outcomes [30]. Even though mortality was adjusted for hypotension at admission by Teixeira et al*.*, possible episodes of hypotension particularly in the field and during transportation may not have been displayed; whereas, this would have been represented by variables of the RISC-II score like acidosis, coagulation or hemoglobin [18].

A limitation of the present study and a general issue of large-scale databases is the risk of incomplete or incorrect data despite various electronic plausibility checks when entering online data, which may have affected the presented results. Evaluation and documentation of source data are subject to interrater variability influenced by training background and local conventions. Patients documented only via the basic data set of the TR-DGU were not considered because no information about surgical procedures is collected for these cases. Possibly, this leads to a bias of higher representation of high-level trauma center in this study since the basic data set is mostly used by smaller hospitals. Nevertheless, the evaluated dataset is still among the largest sets available and provided a sufficient patient number for statistical analyses. The presented findings are only generalizable on cohorts within a comparable health care system and with similar inclusion and exclusion criteria. Notably, as in the NTDB study [14], all patients who died within the first 48 h have been excluded to focus on the effect of splenectomy on secondary brain injury and to control for the self-fulfilling prophecy effect of initial withdrawal of life-sustaining therapy due to high severity of primary trauma. Since inflammatory reaction exhibits temporal changes of cytokine and chemokine concentrations implicating also functional alterations [22], a potential confounding factor is the timing of splenectomy as also addressed by Teixeira et al. [14]. We have, therefore, focused on the role of early splenectomies only. Moreover, this study only looked at discharge mortality and neurologic outcome, and it remains unknown how long-term outcomes will develop at later timepoints. A major limitation is also that early neurologic outcome is based on the Glasgow Outcome Scale, which represents a rather crude assessment tool that was designed to determine long-term outcomes [16]. This scale might not be sensitive enough to detect subtle differences in the early neurological and functional status of a patient and therefore does not allow to investigate the effect of splenectomy on neurological outcome with scientific certainty. Access to scales of more complexity like the Rancho Los Amigos Scale would be advantageous for future studies [31].

The present main results and conclusions of this study are based on a logistic regression model with clear limitations. It is understood that such data are generally to be interpreted with caution and should not uncritically guide towards a change of paradigms without further investigation. Alike, this study cannot fully reject the concept of a detrimental peripheral inflammatory reaction which has previously been shown by experimental data. It seems natural that a direct comparison between a polytraumatized patient and a splenectomy under highly controlled experimental conditions without actual trauma other than TBI is challenging. It needs to be considered, that in the setting of polytrauma, also other injury factors like tissue damage and necrosis [32, 33] or hemorrhagic shock and subsequent massive blood product substitution [34] may drive peripheral inflammation and lead to a systemic inflammatory response syndrome. Despite extensive adjustment for confounding factors, the effect calculated for splenectomy based on the real-life data from a registry presumably still covers both the impact of the intervention itself—in which we are interested and on which the experimental data are based on—and also the critical overall situation which led to the splenectomy. A discrepancy between registry and preclinical studies might, therefore, be due to unknown variables we cannot adjust for as well as to a temporal bias given that we adjusted with the RISC-II score only for data collected at admission. However, a potential beneficial effect on in-hospital mortality seemed not strong enough to be measurable by our methodology. A prospective interventional trial, which would assess this concept in a more controlled fashion, appears not feasible for obvious ethical reasons.

## Conclusions

In a cohort of patients with moderate-to-severe TBI and concomitant splenic injury, patients undergoing splenectomy demonstrate a more severe injury pattern, more compromised hemodynamic status and higher in-hospital mortality than patients without splenectomy. Adjustment for confounding factors reveals that the splenectomy procedure itself is not independently associated with survival. Matched pairs of patients with either splenic preservation surgery or splenectomy demonstrate no differences in mortality.

## Data Availability

The publication guideline of the TraumaRegister DGU^®^, at present, denies external access to raw data captured in the registry.

## Code availability

Statistical operations are reported in the manuscript.
